# Co-producing a multi-stakeholder Core Outcome Set for distal Tibia and Ankle fractures (COSTA): a study protocol

**DOI:** 10.1186/s13063-021-05415-1

**Published:** 2021-07-12

**Authors:** Nathan A. Pearson, Elizabeth Tutton, Alexander Joeris, Stephen Gwilym, Richard Grant, David J. Keene, Kirstie L. Haywood

**Affiliations:** 1 Warwick Research in Nursing, Warwick Medical School, University of Warwick, Coventry, UK; 2 Oxford Trauma and Emergency Care, Kadoorie Centre, Nuffield Department of Orthopaedics, Rheumatology and Musculoskeletal Sciences, University of Oxford, Oxford, UK; 3 Trauma and Major Trauma Centre, Oxford University Hospitals NHS Foundation Trust, John Radcliffe Hospital, Oxford, UK; 4 AO ITC, Clinical Science, AO Foundation, Davos, Switzerland; 5 National Institute for Health Research, Applied Research Collaboration, Coventry, UK; 6 Warwick Medical School, User Teaching and Research Action Partnership, Coventry, UK; 7 Nuffield Department of Orthopaedics, Rheumatology and Musculoskeletal sciences, Fragility Fracture Network, University of Oxford, Oxford, UK

**Keywords:** Distal tibia, Malleolar, injury, Ankle fracture, Trauma, Core outcome sets, Interviews, Delphi study, Systematic review

## Abstract

**Background:**

Ankle fracture is a common injury with a strong evidence base focused on effectiveness of treatments. However, there are no reporting guidelines on distal tibia and ankle fractures. This has led to heterogeneity in outcome reporting and consequently, restricted the contribution of evidence syntheses. Over the past decade, core outcome sets have been developed to address this issue and are available for several common fractures, including those of the hip, distal radius, and open tibial fractures. This protocol describes the process to co-produce—with patient partners and other key stakeholders—a multi-stakeholder derived Core Outcome Set for distal Tibia and Ankle fractures (COSTA). The scope of COSTA will be for clinical trials.

**Methods:**

The study will have five-stages which will include the following: (i) systematic reviews of existing qualitative studies and outcome reporting in randomised controlled trial studies to inform a developing list of potential outcome domains; (ii) qualitative interviews (including secondary data) and focus groups with patients and healthcare professionals to explore the impact of ankle fracture and the outcomes that really matter; (iii) generation of meaningful outcome statements with the study team, international advisory group and patient partners; (iv) a multi-round, international e-Delphi study to achieve consensus on the core domain set; and (v) an evidence-based consensus on a core measurement set will be achieved through a structured group consensus meeting, recommending best assessment approaches for each of the domains in the core domain set.

**Discussion:**

Development of COSTA will provide internationally endorsed outcome assessment guidance for clinical trials for distal tibia and ankle fractures. This will enhance comparative reviews of interventions, potentially reducing reporting bias and research waste.

## Background

Fractures are a common problem with incidence rates in the UK reported to be 73.3 per 10,000 in adults aged 18–49 years, increasing to 116.3 per 10,000 in adults aged 50+ years [1]. Recent evidence indicates that 14% of hospital fracture admissions in England were for distal tibia and ankle fractures [2]. Lower-limb fractures are associated with both short and long-term disability and pain [3, 4]. Evidence suggests that recovery can be slow [5] with substantial variation in how quickly or successfully patients return to their preinjury lives [5, 6]; many patients report experiencing prolonged disability [7]. However, evidence exploring the recovery and impact of such injuries is limited. Growing evidence suggests that an ankle fracture can affect physical, social and psychological functioning with indications of fatigue, depression, anxiety, and disturbed sleep [8]. However, due to inconsistent outcome selection and reporting, it is difficult to collate and synthesise evidence to scrutinise these findings [9–11].

Evidence syntheses are essential for collating and determining the strength of evidence for interventions, informing, and advancing healthcare provision. However, such evidence syntheses (e.g. meta-analysis) rely on homogeneity in outcome selection and reporting to draw evidence together [12]. The need and benefits of uniformity in outcome selection and reporting have been well described [13–16], including the following: (i) increased relevance of outcomes included in trials (to healthcare services users and professionals); (ii) enhanced homogeneity in outcome reporting between studies, thus strengthening meta-analyses through wider inclusion of research studies; (iii) reducing outcome reporting bias; and (iv) reducing research waste [16].

Significant progress has been made towards developing reporting guidance across a range of fractures, with core outcome sets in development or available for hip [17], distal radius [18], open lower limb [19], and shoulder disorders (including proximal humeral fractures) [20]. However, there is currently no guidance for distal tibia and ankle fractures. In a 2012 Cochrane review of rehabilitation for adults with ankle fractures, researchers highlighted that clinical and statistical heterogeneity between studies was a substantive challenge when conducting meta-analyses [9]. This issue has persisted with subsequent reviews examining return to sport in adults [10] and managing low risk ankle fractures in children [11] also identifying the challenge of heterogeneity in outcome selection and reporting and its detrimental effect on conducting evidence synthesis. The need for a core outcome set for distal tibia and ankle fractures is clear and has been recently recommended to increase outcome reporting homogeneity in ankle fracture research [21]. Currently, there is work ongoing to develop a core outcome set for ankle fractures specifically for children [22] but no guidance for adults.

A Core Outcome Set (COS) is an agreed minimum number of outcomes that should be measured and reported in all trials for a specific clinical area [16]. Two key development stages are described [23–25].

1. Clarifying the outcome domains that matter to key stakeholders and should be minimally assessed in future clinical trials (Core Domain Set; CDS).
2. Confirming the assessment method(s) for each outcome domain (Core Measurement Set; CMS).

Current best practice guidance [24, 25] describes a mixed-methods approach, integrating evidence from literature reviews, patient interviews, and active engagement with key stakeholders to reach consensus. Additionally, establishing advisory groups with key stakeholders is recommended and widely described [26–29], contributing to improved COS uptake [16]. Similarly, public involvement groups can enhance the full research process, including identification of unique outcomes for incorporation within the COS [16, 30].

This protocol describes the process for co-producing a multi-stakeholder derived Core Outcome Set for distal Tibia and Ankle fractures (COSTA).

## Methods

**Fig. 1:**
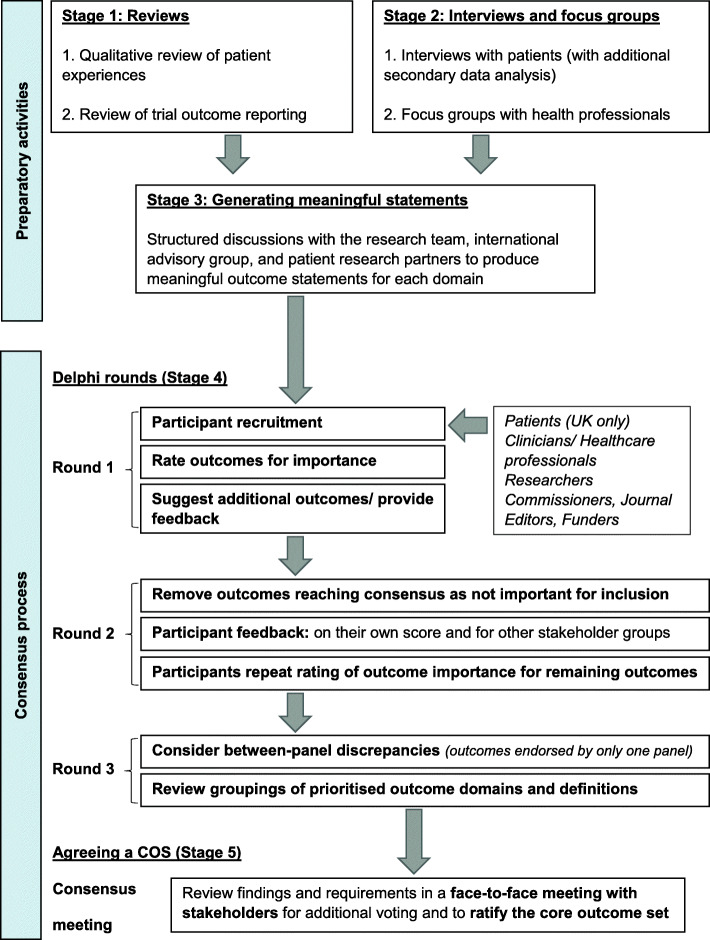
Flow diagram illustrating the process of developing COSTA

A five-stage approach will be adopted (Fig. 1) [24, 25]. The first three stages include preparatory activities to inform the generation of potential outcome domains for consideration in the e-Delphi study: systematic reviews of existing qualitative studies and outcome reporting in randomised controlled trial studies (stage 1) qualitative interviews (including secondary data) and focus groups with both patients and healthcare professionals to explore the impact of ankle fracture and the outcomes that really matter (stage 2) and generating meaningful outcome statements with the research team, international advisory group, and patient partners (stage 3). A multi-round, international e-Delphi study will seek to achieve consensus on the Core Domain Set (CDS) for the distal tibia and ankle fracture core outcome set (COSTA) (Stage 4) and, finally, a consensus meeting to agree a Core Measurement Set (CMS) and recommend the COS for distal Tibia and Ankle fracture (COSTA) (Stage 5). The COSTA project is registered with the Core Outcome Measures in Effectiveness Trials (COMET) initiative (http://www.comet-initiative.org/Studies/Details/1488).

### Aim

A protocol for the development of a core outcome set for distal tibia and ankle fractures (COSTA) for use in clinical trials.

### Objectives

1. To develop a long list of important outcome domains, incorporating the perspectives of multiple stakeholders (researchers, patients, healthcare professionals) (stages 1 and 2).
2. To generate meaningful statements for identified outcome domains (stage 3).
3. To achieve consensus on the core domain set for COSTA (stage 4).
4. To achieve consensus on a core measurement set for COSTA (stage 5).

### Design

Three groups will be actively involved in the COSTA project:

1. *Core research team*: consisting of methodologists, clinicians, and a patient partner, will meet monthly to discuss project design issues and progress.
2. *International advisory group*: consisting of methodologists (core outcome set, health measurement), clinicians, and researchers with experience in distal tibia and ankle fractures and clinical trials, with representation from funding bodies and journals. This group will meet every 6 months to discuss COSTA progress.
3. *Patient and public involvement (PPI) group*: will be established to work collaboratively with the core research team in delivering the project. Their involvement will ensure that COSTA is relevant and acceptable to distal tibia and ankle patients. Regular meetings and email communication will be initiated to achieve and support active engagement.

### Scope

The purpose of COSTA is to provide outcome selection and reporting guidance for:

*Research or practice settings*: For adoption in clinical trials.

*Health condition*: Fracture of the distal tibia and ankle.

*Population*: Adults (aged 18 years or older with closed growth plates) who have experienced a distal tibia (tibia, distal end segment type 43) and ankle fracture (malleolar segment type 44) as defined by the AO/OTA classification [31].

*Intervention*: Any surgical or non-surgical intervention including physical rehabilitation.

### Incorporating patient and public involvement (PPI) into COS development

A COSTA-specific PPI group will be established from existing PPI networks. Members will include individuals who have experienced distal tibia and ankle fractures or been involved in caring for someone following an ankle fracture. Evidence suggests that failure to incorporate patients can lead to important outcomes being overlooked [32]. Moreover, active PPI can enrich the research process through improved outcome identification [33, 34] and greater participant retention in key stages of COS development, e.g. e-Delphi surveys [35]. Active collaboration with PPI members as patient research partners in the research process will ensure that outcomes that are important and relevant to patients are reflected in the COS [30].

A ‘co-production’ approach to COS development will be adopted [36], underpinned by the COMET People and Patient Participation, Involvement and Engagement (PoPPIE) guidance [37] and the six UK standards for public involvement [38]. Our patient research partners will have roles that are comparable to the research team but without the day-to-day responsibilities of running the project. They will be supported to contribute throughout the study, including shaping ethics applications, study materials (e.g. interview schedules), data analysis and interpretation, reviewing, modifying, and generating meaningful statements for the outcomes list and ratifying the core domain set. Their involvement will be reported using the Guidance for Reporting Involvement of Patients and the Public (GRIPP2) short-form checklist [39].

### Stage one: reviews of outcome reporting in clinical trials and of patient experiences of recovery

We will conduct two systematic literature reviews to identify reported outcomes in (1) intervention randomised control trial (RCT) studies and (2) qualitative studies exploring the patient experience and recovery following an ankle fracture. Both reviews will use the Preferred Reporting Items for Systematic Reviews and Meta-Analyses [40] guidance to support transparency in reporting.

#### Review of trial outcome reporting

This review will support the development of an outcomes list that incorporates what is important to researchers. Searches will be conducted across five databases (from 2000 to 2021): Medline (OVID), EMBASE (OVID), PsycINFO (OVID), CINAHL, and AMED. Additionally, three trial registries (ISRCTN, ICTRP, and ClinicalTrials.gov) and the Cochrane Central Register of Controlled Trials (CENTRAL) will be searched. One experienced reviewer (NP) will screen titles and abstracts and determine whether they meet eligibility criteria (Table 1); a 10% subset will be double assessed by another experienced reviewer (KH) to check for accuracy and consistency. Any disagreements will be resolved through discussion and if necessary, a third reviewer (ET) will be consulted. NP and KH (10% subset) will extract information which will include the study reference, design, population and sample size, interventions studied, and outcomes reported (e.g. health, clinical, social, economic) and measurement approaches (e.g. patient or clinician reported outcome). Additionally, the review will also examine clarity of reporting and extract information on whether used measures are reproducible, include appropriate references, and if they have been modified for the study. Outcomes will be grouped using the World Health Organisation (WHO) International Classification of Functioning, Disability and Health model (ICF) [41].

**Table 1:** Eligibility criteria for inclusion in reviews

	Inclusion criteria	Exclusion criteria
**Trial outcome reporting**	Randomised clinical trials examining non-pharmacological interventions for adults with an ankle fracture	Article reports multiple lower limb fractures that include the ankle (e.g. foot, tibia), or paediatric patients
	Available in English, full text and published in a peer-reviewed journal	Trial focuses on screening or diagnostic methods
		Study is an animal or cadaver study
		Publication is a conference proceeding, editorial or available as an abstract only
**Reports of patient experiences**	Conducted with people who have experienced distal tibia or ankle fracture	Conducted with healthcare professionals
	Have used qualitative research methods to explore experience of injury and recovery from distal tibia or ankle fracture	Studies using quantitative methods only
	Mixed method studies with a separately analysed and reported qualitative component	Publication is a conference proceeding, editorial or available as an abstract only
	Article available in English, full-text and published in a peer-reviewed journal	

#### Review of qualitative reports of patient experiences of recovery following ankle fracture

This review aims to examine and synthesise evidence from existing qualitative studies exploring patient experience of injury and recovery from distal tibia or ankle fracture, ensuring relevant outcomes to patients are reflected in the developing outcomes list. Additionally, this review will contribute to our wider understanding of ankle fracture experience and recovery.

Searches will be conducted across five databases (from 2000 to 2020): Medline (OVID), EMBASE (OVID), PsycINFO (OVID), CINAHL, and AMED. One experienced reviewer (NP) will screen titles and abstract to determine eligibility with a 10% subset double-assessed by a second experienced reviewer (ET) to check for accurate and consistent application of the eligibility criteria (Table 1). Disagreements will be resolved, where necessary, through discussion or in consultation with a third reviewer (KH).

Data will be extracted and analysed by NP and ET and managed using NVivo software. Data will be analysed using thematic synthesis [42] to elucidate higher-order analytical themes; this will supplement the extraction of important outcomes. Outcomes will be grouped using the WHO ICF framework [41] and incorporated into the developing outcomes list.

### Stage two: Understanding experiences and ‘what matters’ to patients and healthcare professionals

#### Part A: Interviews with patients

We will conduct semi-structured interviews with a purposive sample of up to 30 individuals who have experienced distal tibia and ankle fractures. This sample size will allow for a range of views to be expressed across injuries (distal tibia, ankle) and age groups (younger and older adults) to be captured. Interviews will be guided by an interview schedule co-produced between the research team and patient partners (Table 2) and are anticipated to last up to one 1 h. All interviews will be audio-recorded and transcribed verbatim. Notes will be taken during and after interview to support analysis, interpretation, and reflexivity.

**Table 2:** Patient co-produced interview questions and prompts

Interview questions and prompts	
**The two key questions will be:** What has it been like for you since you injured your ankle? Prompts: Tell me more about that, how did you feel, what did you think? What is important to you about your recovery from the fracture? Prompts: What is most important to you? What are you hoping for? Other questions may be useful to guide the interview such as:	
Could you tell me about how you fractured your ankle?	
How did you/ are you managing with the injury? - Work - Personal and social life - Feelings and mood	
What was most difficult time for you?	
How are you feeling/ what are you thinking/ about your recovery?	
How has your injury impacted on you?	
Are there things you could do before, but you can’t now?	
Did/ do you have any worries or concerns about your injury or recovery?	

Sampling criteria will include their treatment strategy (operative or non-operative), type of injury, time since injury, and geographic location. Recognising that there is a bimodal incidence of ankle fractures in adults by age, with younger patients often experiencing high energy fractures and older patients more often experiencing low energy fractures [43], sampling will ensure a range of ages are included to ensure a breadth of perspectives are captured in interviews. Participants will be aged 18 years or older, will have experienced distal tibia and ankle fractures within the last 9 months, and be able to communicate (written and verbally) in English. Participants will be identified through clinical lists maintained by their local hospital trauma department. Given the evolving situation with COVID-19, interviews will either be conducted face-to-face, virtually (via webcam) or by telephone, depending on safety guidance. Patients will be unknown to the researcher prior to interview.

The interviews will draw on interpretative phenomenological analysis (IPA) as a methodological framework [26], an analysis process developed specifically to provide methodological direction for research that focuses on understanding the lived experience of others [26]. The analysis consists of two elements:

1. A thematic analysis [44], drawing on the impact triad of severity, importance, and self-management [45], will be conducted to develop a framework of meaningful patient outcomes. These outcomes will be mapped to the ICF framework [41] and contribute to the developing outcomes list (from the systematic reviews).
2. A separate IPA will be conducted on a homogeneous subset of up to 10 interviews. This will make a separate contribution to the evidence base by developing our understanding of the lived experiences of patients with distal tibia and ankle fractures and their recovery. The analysis will be inductive and continuous, following the process described by Smith, Flowers, and Larkin [46].

Interviews will be conducted and coded by NP, ET, and KH, none of whom are involved in routine patient care. All researchers are experienced in conducting interviews and have used IPA and thematic analysis. NVivo software will be used to manage the data. NP is experienced in mixed methods, a male Research Fellow with a PhD in Health Sciences, and a background in Psychology and Cognitive Neuroscience. ET is an experienced, qualitative, female researcher with a PhD in Health Sciences and a focus on traumatic orthopaedic injuries. KH is a female with a DPhil in Health Sciences and Clinical Evaluation; she is a mixed methods researcher with experience across a range of patient populations.

Rigour will be guided by the principles proposed by Yardley [47]. These criteria reflect sensitivity to context, commitment and rigour, transparency and coherence, and impact and importance. Sensitivity to context will be demonstrated through consideration of relevant literature, theoretical understanding and understanding the perspective of participants. Commitment and rigour will be demonstrated through in-depth engagement with the topic, thorough data collection and the depth of analysis. Transparency and coherence will be demonstrated through the detailed accounts from participants, reflexivity, and the production of a clear audit trail that captures key quotes that inform theme derivation. Finally, impact and importance will be demonstrated through development of our theoretical and practical understanding of fracture experience and recovery.

#### Secondary data analysis of existing interview data

A secondary analysis of existing interview data, garnered from a qualitative sub study of the “Ankle Fracture Treatment: Enhancing Recovery” (AFTER) [48] study, will be conducted (ET). The main study included 60 adults, aged 50 years and above, who had sustained an ankle fracture and consented to take part in a study of progressive functional exercise versus best practice advice. The qualitative study explored the lived experience and 6-month recovery in twenty patients. Important outcomes will be extracted from this data as part of the ongoing phenomenological analysis. The AFTER study is wholly independent of the COSTA initiative and will be reported separately by the respective authors (DK, ET).

#### Part B: Focus groups or interviews with healthcare professionals

Given the evolving circumstances and current pressures on healthcare professionals regarding COVID-19, the conducting of focus groups may not be feasible at the point of study commencement. Consequently, alternative arrangements have been developed which would use interviewing as per the approach taken in Part A with patients. Here, we will explore the experiences of professionals working with or treating ankle fracture patients before reviewing and potentially adding to the outcomes list derived from the systematic reviews. Whilst the benefit of group discussion and sharing experiences may be lost using an interview approach, they will ensure that the healthcare professional perspective is incorporated into the developing outcomes list.

Should it be possible to host focus groups, up to four will be held and co-facilitated by members of the research team with experience in conducting focus groups. Previous experience suggests that focus group attendance tends to be difficult to predict. Current guidance recommends between 5 and 12 participants are required for an effective focus group discussion. Therefore, this study will seek to recruit at the upper end (*n* = 12) of this guidance to accommodate likely attrition [49–51]. Professionals will be purposively sampled based on their professional role (e.g. clinicians, occupational therapists, physiotherapists, nurses). It is anticipated that the focus group will take up to 1 h, including a short 10-min comfort break. To participate, healthcare professionals will have experience working within a trauma setting, including with patients who have experienced relevant fractures to the study (distal tibia or ankle). Professionals will be identified and approached by site principal investigators, snowballing, and personal contacts.

Data will be analysed using thematic analysis [44] to identify key outcomes and themes as per the process described in Part A interviews. The findings of the focus groups/ interviews will support identification of distal tibia and ankle fracture outcomes that are considered important to healthcare professionals. The process of ensuring rigour and transparency will again follow the principles proposed by Yardley [47].

### Stage three: Generating meaningful outcome statements

The long list of outcomes generated in stages 1 and 2 will be discussed with members of the core, international advisory, and PPI groups. Working collaboratively with all members, outcome statements will be generated for each outcome domain and mapped to the World Health Organisation (WHO) International Classification of Functioning, Disability and Health model (ICF) [41]—a structured and widely recognised outcomes framework that will support greater transparency and consistency in the language used to describe potential outcome domains [52].

A face-to-face (or digitally hosted) meeting will be scheduled across one full day (if possible) or alternatively, two half-day sessions, and materials sent to participants in advance of the meeting for their consideration. These materials will include research findings (from reviews and qualitative data) and a questionnaire for participants to complete and return in advance of the meeting, listing all outcomes with potential definitions. When completing the questionnaire, participants will consider outcome domains, definitions, and whether any important outcome domains are missing. The meeting will be structured using a modified nominal group technique to facilitate group discussions. This will consist of three stages:

1. *Reviewing the research findings*: work completed will be discussed; the results of the pre-meeting survey will be discussed.
2. *Refining outcome priorities*: the list of outcome domains will be reviewed and refined to reduce repetition, ensure appropriate grouping of outcome domains, and review the mapping of outcome domains to the WHO-ICF framework.
3. *Producing meaningful outcome statements*: we will consider, and where necessary, revise outcome statements for each outcome domain through discussion to achieve a list of outcome domains with meaningful statements.

The revised outcome domains list and meaningful statements will be written in plain English, using both open and closed-format questions, forming the basis for an on-line e-Delphi questionnaire (stage 4). The questionnaire will be piloted with the core team, PPI partners, and researchers naïve to the study (*n* = 10).

### Stage four: Achieving outcome prioritisation and core domain confirmation in an international, multi-stakeholder e-Delphi study

Delphi studies utilise a process of sequential questionnaire completion and feedback to establish expert consensus between a panel of experts [53]. To ensure that the COSTA reflects the perspectives of experts in the field of tibial and ankle fracture, two panels will be defined: (1) patients who have experienced a distal tibia or ankle fracture and (2) health professionals and researchers who are active in this field, representative of their professional groups, and well-positioned to implement the COSTA recommendations [54].

Consensus or accepted standards for sample sizes for Delphi studies are currently lacking [53], with expert panel sizes described in COS development ranging from 15 [55] to over 200 panellists [56]. We refer to recent examples of Delphi studies where pre-identified expert panels of between 60 and 70 panellists per panel are described [57–59]. This will facilitate a wide expert view and accommodate for attrition across rounds. Eligibility criteria for panellists are presented in Table 3.

**Table 3:** Eligibility criteria for participants in the e-Delphi

	Inclusion criteria	Exclusion criteria
**Generic criteria (all)**	Aged 18 years or older	Unable to access a computer or digital device for the duration of the study
	Willing to participate in a multi-round online Delphi study	
	Proficient in written English	
**Expert panel 1 (patients)**	Has experienced a fracture of the distal tibia/ankle within the last 2 years (at the point of contact)	Fracture involved multiple sites defined as outside of the distal tibia and ankle (AO criteria [31])
**Expert panel 2 (professionals)**	Has experience working, or conducting research, with ankle fracture patients	Limited experience (less than 9 months) working in fracture care and no experience of working with distal tibia and ankle fractures

*Expert panel 1*: We will identify patients—adults aged 18 years and over who have sustained a fracture of the distal tibia or ankle within the 2-year period of the start of the e-Delphi—as per the recruitment approach specified in stage two. This will be a UK-based sample. A ‘pre-agreed’ list of participants will be established and invited to participate in the e-Delphi.

*Expert panel 2*: A selected group of international health professionals (clinicians / surgeons, physiotherapists, occupational therapists, nurses), and researchers (trialists, reviewers, measurement experts) known to be actively involved in delivering fracture care, or in fracture research of relevance to distal tibia and ankle fractures, will be identified through national and international professional networks (e.g. AO Trauma Network) and societies (e.g. the British Orthopaedic Foot and Ankle Society, Association of Foot and Ankle Physiotherapists), published research and involvement in clinical trials. Recruitment approaches will be supplemented by snowballing and personal contacts. Potential participants will be invited by e-mail to consider participation in the study. A pre-agreed list will be established and invited to participate in the e-Delphi.

#### Modified e-Delphi method

The modified e-Delphi will be conducted using the COMET DelphiManager software (University of Liverpool). It will consist of three sequential rounds with the same group of panellists: participants completing round 1 will be eligible to complete round 2, and those completing round 2 will be eligible to complete round 3. Participants will have up to 2-weeks to complete each round, with reminders sent after 1 week and again 24 h before the round is closed. Data will be analysed using descriptive statistics and presented using measures of central tendency, and as graphs, where necessary. Missing data will be examined across each outcome domain to identify potential patterns in non-response (e.g. by panel, or other characteristics). If an item-level pattern is observed (i.e. > 10% of responses are missing), we will check this against qualitative feedback provided by participants.

*Round 1*: Participants will be invited to rate the relative importance of each outcome domain for ‘inclusion in future distal tibia and ankle fracture research studies’ using a nine-point numeric rating scale (range: 1–3 ‘not at all important’, 4–6 ‘uncertain’ and 7–9 ‘very important’). An additional option ‘unable to rate’ will also be provided. Participants can elaborate on their decisions, providing additional qualitative comments and feedback for consideration in subsequent rounds. Evidence suggests that attrition can be reduced with such active engagement [53, 60].

A reduction in the number of outcome domains is a key objective of the Delphi study, seeking to achieve consensus on a minimum number of outcome domains (core domain set) for the COS. Therefore, a bespoke grading system, described in the development of a COS for migraine (COSMIG), will be adopted to provide greater clarity where participants from different sub-groups either agree or disagree in their judgements [61]. This approach defines clear criteria and decision rules for handling differing levels of consensus e.g. little or no consensus (grade C or D), uncertainty (grade A and B), and clear consensus (grade A** and A*) per outcome domain. Only outcome domains judged most favourably by one or both expert panels will be included in round two (Table 4).

**Table 4:** Grading system for determining consensus in round 1 of the e-Delphi study

Grade	Criteria for judging agreement	Decision rule
A**	Median rating is 9 for both expert panels	Include in round 2
A*	≥ 70% of respondents in each panel rate an outcome domain ≥ 7	Include in round 2
A	Median rating for an outcome domain is ≥ 7 for both expert panels	Include in round 2 if one of the panels achieves a median score of 9 OR qualitative evidence supports further consideration
B	Median rating for an outcome domain is ≥ 7 for only one expert panel	Include in round 2 if this group achieves a median score of 9 OR qualitative evidence supports further consideration
C	Median rating for the two panels combined is ≥ 4 and ≤ 6, and the median rating for no single panel is ≥ 7	Exclude from round 2 (unless strong qualitative evidence supports further consideration)
D	Median rating for the two panels combined is ≥ 1 and ≤ 3, and the median rating for no single group is ≥ 7	Exclude from round 2 (unless strong qualitative evidence supports further consideration)

*Round 2*: Responses to round one will be summarised (individual and group median scores for each outcome). Qualitative feedback will inform the inclusion of additional or edits to existing outcome domains.

Further prioritisation will be sought by inviting panellists to allocate ‘points’ to illustrate how important they feel an outcome domain is for inclusion in the core domain set. Each outcome domain will be assessed using an 11-point numeric rating scale, where 0 is not a priority, and 10 an absolute priority. A maximum of 70 points can be spent, with a maximum of 10 points can be assigned to any one outcome domain.

The sums of priority ratings will be calculated (per sub-panel and combined), supporting the identification of both the top 10 and top 50% of prioritised outcome domains. These will be discussed by the core research team. Outcome domains will be retained as they are, or if considered to be similar concepts of health, grouped into a new higher order of ‘meaningful’ outcome domains [61, 62].

*Round 3*: Participants will be asked to consider if they are happy with a series of decisions informed by earlier rounds. First, they will be advised of the top 50% of prioritised outcome domains from round 2. They will then be advised of any between-panel discrepancies. Where outcome domains are prioritised by just one panel, respondents will be asked to consider if they should be included in the prioritised list (Yes/No); outcome domains voted for inclusion by ≥70% of respondents will be included. Finally, respondents will be asked to specify if they agree (Yes/No) with the grouping of prioritised outcome domains, and the meaningful outcome domains and definitions. An option to provide additional comments will be available. A frequency distribution of responses will be computed.

### Achieving a core domain set

It is anticipated that a core domain set will be achieved by the end of round 3. Whilst there is no agreed ‘ideal’ number of core domains, to be feasible and acceptable, a smaller core domain set is recommended (i.e. fewer than 8 outcome domains) [63]. However, numerous examples of COS development have required further consensus activities to clarify and ratify findings from the Delphi process [20, 27, 61]. Recent protocols to develop core outcome sets have also outlined plans for consensus meetings to ratify a core domain set [64, 65]. Should a core domain set not be achieved following the e-Delphi, then the findings will be taken to a consensus meeting for final voting and ratification (stage 5) before seeking consensus on a core measurement set.

### Stage 5: Consensus meeting

The aim of this meeting will be to confirm the core domain set (stage 4), agree a core measurement set (CMS) and recommend the COS for distal Tibia and Ankle fracture (COSTA). Outcome domains that are considered important but do not have an available measurement approach will not be included within the COS.

We will invite health professionals and patients who participated in the e-Delphi study to take part in this consensus meeting. In advance of the meeting, participants will be provided with an information pack outlining the objectives of the meeting—this will include the CDS and short-listed outcome measures to be considered for the CMS.

#### Developing the core measurement set

In advance of the meeting, we will identify available guidance or existing consensus on how best to measure short-listed outcome domains. Where there is no existing consensus for potential outcome measures, the core research team will review key evidence sources to determine measurement quality [66], acceptability, and feasibility.

The meeting will begin with an overview of the e-Delphi and the prioritised outcome domains, with accompanying information on the evidence underpinning identified measures. Participants will be asked to consider ‘placement’ of the outcome domains within the final COS [61, 62]:

1. Core ‘inner’ circle: outcome domain is clear with an acceptable, method of assessment.
2. Middle circle: whilst important, the inclusion of this outcome domain in all trials is not feasible (e.g. lack of available assessment method).
3. Outer circle: whilst important, the current available evidence for the conceptualisation of the outcome domain or assessment method is limited.

The meeting will be structured into three parts:

i. Initial presentations to the large group will be followed by facilitated small-group discussions with a mixed group of stakeholders. Groups will discuss each prioritised outcome domain and potential method of assessment, considering evidence of quality, acceptability, feasibility, and importance. Findings between groups will then be shared to facilitate further discussion.
ii. At the end of each small-group discussion, participants will anonymously complete a questionnaire to confirm the inclusion of each outcome domain (Yes/No/Don’t know) and their preferred method of assessment (selecting from a short-list). Agreement will be defined as ≥ 70% participants endorsing an outcome domain and/or method of assessment.
iii. A whole group discussion will be facilitated to discuss outcome domain priorities or assessment methods. Where there is agreement, no further discussion will be required. Subsequent discussions will focus on areas of disagreement and where further refinement is required. Finally, participants will be asked to anonymously vote electronically to confirm ‘placement’ of all core domains (inner, middle or outer circles), and, where previously not confirmed, the preferred method of assessment.

Written notes will be taken throughout the sessions, and the voting will be recorded.

The outcome of this consensus meeting will be to ratify a COS for distal tibia and ankle trials, identifying both the core outcome domains that should be minimally assessed, alongside evidence-based recommendations on current best methods for assessment.

### Dissemination

In line with recommendations, ongoing work will be necessary to maximise the uptake and implementation of the COS [16]. We will seek to actively address this challenge through our close active collaboration with our PPI group and engagement with our international advisory group.

Participants in the research will be informed of project outcomes through the sharing of a summary document following completion of each stage. This will be shared directly with participants via email. COS users (including funders and journal editors) will be reached through a range of methods, including wide dissemination through publications and both national and international conference presentations. Additionally, AO Trauma’s international reach through their leading expertise in trauma will enable the team to directly inform the international trauma community about COSTA.

## Discussion

Currently, outcome reporting guidance for distal tibia or ankle fractures is lacking, despite the need for such guidance being highlighted in recent reviews [9–11]. The development and uptake of a core outcome set for tibia and ankle fractures (COSTA) will ensure that outcomes viewed as important to key stakeholders are included in clinical trials, supporting the development of an evidence base that can be synthesised and better examined to support healthcare decisions.

A limitation of the COS is that it will rely on a UK sample of patients throughout. This is due to project time constraints which are amplified by COVID. However, systematic reviews of international qualitative and quantitative literature will be used to inform an initial long list of potential outcome domains.

A well-developed COSTA will address the current challenges associated with heterogeneity in outcome selection and reporting in ankle fracture research [9–11] through improving opportunities for evidence synthesis, creating opportunity for comparative reviews of care provision across different fracture types, reducing reporting bias, and reducing research waste [13–15]. To ensure these benefits are brought to fruition, ongoing work will be necessary to ensure the uptake and implementation of COSTA. This will involve maximising dissemination of the COS and highlighting its value in application for both clinical practice and research.

## Trial status

Protocol version number: 1.0

Protocol date: March 8, 2021

Recruitment start date: delayed due to COVID

Planned recruitment end date: January 2022

## Data Availability

Data from this study will be accessible to the research team, advisory group, and patient research partners.
